# Socio‐economic predictors and between‐hospital variation in permanent stoma rates after segmental resection of colorectal cancer. A population‐based register study

**DOI:** 10.1111/codi.70153

**Published:** 2025-07-09

**Authors:** Søren Rattenborg, Sören Möller, Erik Frostberg, Hans Bjarke Rahr

**Affiliations:** ^1^ Department of Surgery Vejle Hospital, University Hospital of Southern Denmark Vejle Denmark; ^2^ Institute of Regional Health Research University of Southern Denmark Odense Denmark; ^3^ Open Patient Data Explorative Network, Odense University Hospital Odense Denmark; ^4^ Department of Clinical Research University of Southern Denmark Odense Denmark; ^5^ Colorectal Cancer Center South, Vejle Hospital, University Hospital of Southern Denmark Vejle Denmark

**Keywords:** anastomosis, colorectal cancer, permanent stoma, socio‐economic predictors, surgery

## Abstract

**Aim:**

Annual reports on colorectal cancer (CRC) in Denmark reveal marked variations between hospitals in the rates of permanent stomas. The objective of this study was to explore whether socio‐economic factors and the treating hospital were independent predictors of creating a permanent stoma rather than an anastomosis in segmental bowel resection for CRC.

**Method:**

This was a register‐based national cohort study comprising patients with CRC stratified into four subpopulations according to area of segmental resection: right colon, sigmoid colon, upper third of rectum and mid third of rectum. Primary outcome was anastomosis or permanent stoma, as indicated by the surgeon. We employed multiple logistic regression analyses, adjusting for demographic, performance, lifestyle, comorbid and perioperative factors.

**Results:**

The right colon had insufficient permanent stoma cases for regression analyses. The remaining three groups comprised a total of 14,113 patients. The rates of permanent stoma in both the upper rectum and the mid‐rectum increased with lower household income quartiles. Odds ratios for the lowest income quartiles were 2.1 (95% CI: 1.36–3.13) for the upper rectum and 1.7 (95% CI: 1.25–2.18) for the mid‐rectum compared with the highest household income quartile. Educational level and cohabitation were non‐significant in the adjusted regressions. We found marked between‐hospital variation in permanent stoma rates, compared to the largest hospital, in both sigmoid colon (up to 2.17), upper rectum (up to 4.0) and mid‐rectum (up to 4.6) after adjustment (*p* < 0.01).

**Conclusion:**

A permanent stoma after elective resection of cancer in the upper two‐thirds of the rectum was associated with lower household income. There were marked between‐hospital variations in permanent stoma rates after resection of the sigmoid colon and the upper two‐thirds of the rectum in CRC, even after adjustment for relevant covariates.

**What does this paper add to the literature?**

This paper showed an association of socio‐economic factors with permanent stoma rates after elective colorectal cancer surgery. Income level, but neither education nor cohabitation, was significantly associated with permanent stoma rates after sigmoid and rectal cancer surgery. A marked variation in practice between hospitals was found, despite adjustment for relevant covariates.

## INTRODUCTION

In 2022, around 4,000 individuals in Denmark were diagnosed with colorectal cancer (CRC) [1]. Nearly 80% underwent surgery, which remains the primary curative treatment for CRC. Following resection of the tumour‐bearing bowel segment, surgeons face the decision of either establishing a permanent stoma or restoring bowel continuity through an anastomosis, contingent on technical feasibility. The decision is based on national guidelines [2] and considers various factors, such as disease‐ and patient‐related risk factors for complications [3], intra‐operative feasibility assessments and, notably, the patient's preferences and functional status. While most patients express a preference to avoid a stoma whenever possible, conflicting results arise from studies comparing the quality of life with a stoma to that with an anastomosis [4, 5, 6, 7].

The percentage of patients receiving a permanent stoma post‐CRC surgery varies between hospitals. For example, in 2022, the rate of preserved bowel continuity after rectal cancer resections in Denmark ranged from 41% to 82% [1]. A 2022 annual report from the UK highlighted a wide‐ranging variation in permanent stoma rates in rectal cancer among hospitals/trusts/multidisciplinary teams (MDTs), with a mean of 36% [8]. This may suggest diverse attitudes towards stoma versus anastomosis among hospitals, but it might also be attributed to the between‐hospital variations in case mix that we have previously demonstrated [9].

To our knowledge, no study has investigated whether socio‐economic factors play a role in the choice between anastomosis and permanent stoma, or whether the differences observed between hospitals are systematic, persistent over time and in adjusted analyses.

In a country, like Denmark, with universal free healthcare and national guidelines for cancer treatment, there should be equity among patients in terms of treatment choice and surgical quality. The aim of this study was therefore to investigate whether socio‐economic factors and the treating hospital emerge as independent predictors of permanent stoma rates in elective segmental bowel resection for CRC.

## METHOD

### Design

This study is part of a larger register‐based Danish retrospective cohort project detailed in Rattenborg et al. [9]. The data used in this study were sourced from the Danish Colorectal Cancer Group (DCCG) database [10], the Danish National Health Authority (DNHA) [11], the DNHA's National Patient Registry (NPR) and Statistics Denmark [11].

### Study population

The study population consists of all adults in Denmark diagnosed with first‐time CRC between 1 January 2009 and 31 December 2018 and registered in the DCCG database. Registration is mandatory for all hospitals in Denmark andexceeds 96% [12]. Four specific subpopulations were of interest: (1) right‐sided tumour; (2) sigmoid tumour; (3) high rectal tumour; and (4) mid‐rectal tumour. These tumour locations are prevalent in CRC, and the surgical approach in these cases is straightforward and standardized. We wished to study operations for CRC in which construction of an anastomosis is the standard of care in the absence of any obvious contraindications; therefore, low‐rectal tumours were not included because national guidelines recommend anastomosis only in selected cases of tumour in the lower third of the rectum.

Cancer location at diagnosis was categorized as right colon (caecum, ascending colon, right flexure or transverse colon), sigmoid, upper third of the rectum (11–15 cm from the anal verge) and mid third of the rectum (6–10 cm from the anal verge). In instances where the surgeon's classification of rectal height was absent, preoperative MRI data, when available, was used to determine the height. A recent validation study found that the location of the primary tumour was accurate in 79% of patients. For rectal tumours, distance from the anal verge was accurate in 72% of patients, with low rates of concomitant change between the low, mid and upper thirds of the rectum [12]. Data were retrieved from the DCCG database and also the listed segmental resections:

(1) Right colon: right (extended) hemicolectomy, resection of the transverse colon;

(2) Sigmoid colon: sigmoid resection or Hartmann's procedure of the sigmoid;

(3) Upper third of the rectum: anterior resection, Hartmann's procedure of the rectum, intersphincteric abdominoperineal excision (iAPE) and conventional abdominoperineal excision (cAPE);

(4) Mid third of the rectum: anterior resection, Hartmann's procedure, iAPE and cAPE.

A recent validation study showed that the reported resection in the DCCG was accurate in 90% of the cases [12].

### Eligibility criteria

**FIGURE 1:**
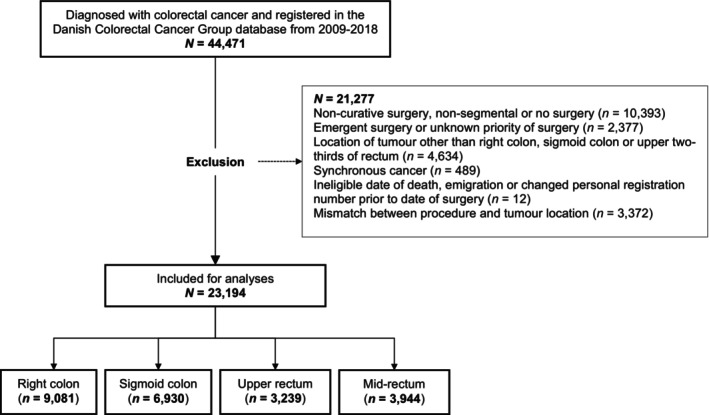
Flowchart of eligibility criteria.

The eligibility criteria, outlined in Figure 1, were as follows:

Inclusion criteria:

- Elective surgery with curative intent;
- Tumour location in the right colon, sigmoid colon or upper two‐thirds of the rectum.

Exclusion criteria:

- Surgery date occurring after the registered date of death or after emigration;
- Change in personal registration number prior to the date of surgery;
- Presence of synchronous cancer;
- Inconsistency between tumour location and the registered surgical procedure (e.g. left hemicolectomy for right‐sided cancer) or substantial deviation from standard treatment (e.g. total colectomy for a localized tumour).

### Primary outcome

The primary outcome variable was the creation of either an anastomosis or a permanent stoma, as defined by Klein et al. [12]. We used the DCCG data hierarchically: patients were categorized as having a permanent stoma if the registered surgical procedure explicitly indicated creation of a permanent stoma (e.g. Hartmann's resection or cAPE) or if the surgeon had documented the creation of a permanent stoma. Otherwise, patients were categorized as having an anastomosis.

### Predictors of interest

#### Hospital of definitive treatment

The hospitals providing definitive treatment were labelled with the letters A to Q. The hospital with the lowest patient volume was labelled “A”, and the hospital with the highest patient volume was labelled “Q”, with labels being allocated before eligibility criteria were applied. Data on patient volume were retrieved from the DCCG database.

#### Socio‐economic factors

Socio‐economic factors, as previously detailed [9], encompassed educational level [13] (short, medium, long, unknown), disposable annual household income adjusted for inflation [14] (1st–4th quartile, unknown) and cohabitation status (cohabiting, living alone, unknown). These three factors are those most applied in healthcare research and provide a broad socio‐economic profile of the population [15]. Furthermore, the data were collected from national administrative registries made available for research from Statistics Denmark.

### Adjustment covariates

The selection of covariates was based on an earlier study [9] and on commonly reported risk factors for adverse outcomes [2, 3].

#### Demographic data, performance and lifestyle

Demographic data included gender (defined by the dichotomy male/female as registered in the personal registration number), age group (<50, 50–64, 65–74, 75–84 or ≥85 years of age), American Association of Anesthesiologists (ASA) score [16] (I–V, unknown) and the World Health Organization (WHO) performance status [17] (0–4, unknown). Lifestyle factors included body mass index based on the WHO classification [18] (underweight, normal weight, overweight, obese, unknown), weekly alcohol consumption in units (0–14, >14 units, unknown; 1 unit of alcohol = 12 g of pure ethanol) and smoking status (never smoker, ex‐smoker, current smoker, unknown). Data were retrieved from the DCCG database.

#### Comorbidity

The aggregate Charlson Comorbidity Index (CCI) score [19] (0, 1, 2, 3+) with updated weights [20] was reported after excluding CRC from the score for the entire cohort. A wide range of comorbidity factors were included in the regression analyses to adjust for possible confounders associated with adverse socio‐economic factors [21]. We included only dichotomous comorbidity variables because these fit better in regression analyses than do CCI scores [22]. The comorbidity variables were based on International Classification of Disease 10th edition (ICD‐10) and Anatomical Therapeutic Chemical Classification System (ATC) codes present up to 10 years prior to diagnosis, as previously outlined [9]. These encompassed nine somatic domains (cardiovascular disease, chronic pulmonary disease, diabetes, dementia, liver disease, kidney disease, chronic nerve disease, other cancer or benign brain tumours, and connective tissue disease) and four psychiatric domains (affective disorders, schizophrenic spectral disease, adult personality and behaviour disorder, and psychoactive substance abuse disorder). Data on ICD‐10 codes were retrieved from NPR via Statistics Denmark, and ATC codes were obtained from DNHA.

#### Disease‐related and surgical factors

The following factors were included: distant metastases at diagnosis (yes, no, unknown); pT4 tumour (yes, no, unknown); whether the patient was discussed in an MDT conference prior to treatment (yes, no, unknown); and neoadjuvant radiotherapy (yes, no, applicable only for rectal cancer). Data were retrieved from the DCCG database.

The year of surgery was included as a continuous variable (2009–2019) because patients diagnosed in 2018 could undergo surgery in 2019. Additional variables were the presence of carcinomatosis intra‐operatively (yes, no, unknown) (missing for the period 2009–2013), clinical signs of tumour perforation (yes, no, unknown) and intra‐operative blood loss (<500, ≥500 mL of bleeding, unknown), as reported by the surgeon. Data were retrieved from the DCCG database.

Registration of a temporary stoma was considered for the anastomosis group (yes, no). Patients were classified as having no temporary stoma when no data on a temporary stoma were available. Data were retrieved from the DCCG database.

### Statistical methods

Analyses were conducted across four groups: right colon, sigmoid colon, upper rectum and mid‐rectum. In clinical practice, the distinction between a low sigmoid tumour and a high rectal tumour is not always clear, prompting the analysis of these two groups as one group initially. However, subsequent examination revealed substantial differences between these two groups, leading to their separate presentation. Descriptive statistics with 95% CI were employed for comparisons between group proportions. Fisher's exact test or the chi‐square test of association was applied on non‐missing categories. Variations between hospitals were presented descriptively. Funnel plots were applied to illustrate differences between hospitals in the proportions of permanent stomas created.

Multivariable logistic regression was performed for the outcome variable permanent stoma, with anastomosis (with or without temporary stoma) as reference. The above‐mentioned covariates were included as independent variables, as shown in the Results section. A global *p*‐value was calculated for between‐hospital variations, based on the regression analyses. Data on the examined predictors were presented as odds ratios with the corresponding 95% CI in the text and in forest plots (based on the regression analyses), adjusting for demographic, lifestyle, comorbidity, disease‐related and perioperative factors. Missing data were mainly because of alterations of data structure in the databases over time and hospital‐specific deficiencies in reporting. No imputation of missing data was performed, and for all categorical variables, missing data were included as an unknown category. Data were securely stored and managed on Statistics Denmark's servers and extracted only after anonymization. All statistical analyses were performed using Stata IC/18 (StataCorp LCC, 4905 Lakeway Drive, TX, USA).

## RESULTS

A total of 23,194 patients were included in this study (Figure 1). Only 67 (0.7%) patients had a permanent stoma in the right colon. Of these 67 patients, 53% had a low level of education, 40% were in the 1st quartile of annual household income and 53% were living alone. The between‐hospital variation in patients with a permanent stoma (as opposed to an anastomosis) in the right colon ranged from zero to 4%. Due to the small number of cases, we excluded the group of segmental resections of the right colon from further analyses.

Table 1 displays the characteristics of 14,113 patients with segmental resections of the sigmoid colon or the upper two‐thirds of the rectum, with or without a permanent stoma. The rates of abdominoperineal excisions (pooled conventional and intersphincteric) were 3.8% in the upper rectum and 18.4% in the mid‐rectum. Patients with a permanent stoma were predominantly older, had higher ASA, performance status and CCI scores, lower educational levels and lower household income. They were more likely to live alone, have distant metastases at diagnosis and to have peroperative perforation and/or high perioperative blood loss.

**TABLE 1:** Characteristics of 14,113 patients with elective segmental resection, with or without permanent stoma for cancer of the sigmoid colon or upper two‐thirds of the rectum (shown as upper rectum and mid‐rectum).

Characteristic	Sigmoid colon			Upper rectum			Mid‐rectum		
	Anastomosis	Stoma	*p*	Anastomosis	Stoma	*p*	Anastomosis	Stoma	*p*
Gender			0.009			0.020			0.442
Male	3581 (90)	409 (10)		1619 (82)	364 (18)		1660 (66)	842 (34)	
Female	2693 (92)	247 (8)		1065 (85)	191 (15)		974 (68)	468 (32)	
Age group (years)			<0.001			<0.001			<0.001
<50	258 (94)	16 (6)		149 (93)	12 (7)		189 (86)	30 (14)	
50–64	1815 (95)	91 (5)		936 (94)	61 (6)		968 (82)	215 (18)	
65–74	2525 (93)	185 (7)		1054 (87)	154 (13)		1083 (71)	453 (29)	
75–84	1424 (85)	251 (15)		499 (67)	245 (33)		380 (45)	470 (55)	
85+	252 (69)	113 (31)		46 (36)	83 (64)		14 (9)	142 (91)	
ASA score			<0.001			<0.001			<0.001
I	1667 (96)	69 (4)		854 (95)	48 (5)		882 (81)	211 (19)	
II	3530 (93)	269 (7)		1541 (84)	293 (16)		1497 (67)	723 (33)	
III	974 (77)	284 (23)		269 (57)	204 (43)		237 (41)	343 (59)	
IV + V	29 (54)	25 (46)		6 (55)	5 (45)		<5 (17)	<25 (83)	
Unknown	74 (89)	9 (11)		14 (74)	5 (26)		<20 (57)	<15 (43)	
WHO performance status			<0.001			<0.001			<0.001
0	2583 (95)	126 (5)		1174 (89)	150 (11)		1146 (73)	415 (27)	
1	715 (84)	135 (16)		212 (68)	100 (32)		177 (46)	208 (54)	
2	145 (66)	75 (34)		30 (45)	37 (55)		<25 (28)	<65 (72)	
3 + 4	31 (54)	26 (46)		5 (36)	9 (64)		<5 (20)	<20 (80)	
Unknown	2800 (90)	294 (^a^0)		1263 (83)	259 (17)		1286 (68)	607 (32)	
Alcohol consumed per week (units^a^)			0.075			0.945			0.016
0–14	4958 (91)	503 (9)		2125 (83)	429 (17)		2061 (66)	1042 (34)	
>14	851 (93)	68 (7)		380 (83)	76 (17)		412 (72)	164 (28)	
Unknown	465 (85)	85 (15)		179 (78)	50 (22)		161 (61)	104 (39)	
WHO body mass index			0.013			0.118			0.017
Underweight	111 (84)	21 (16)		36 (71)	15 (29)		45 (56)	36 (44)	
Normal	2273 (90)	244 (10)		1044 (83)	207 (17)		1105 (68)	529 (32)	
Overweight	2367 (92)	217 (8)		1034 (83)	210 (17)		1002 (69)	458 (31)	
Obese	1242 (92)	114 (8)		462 (84)	91 (16)		402 (64)	230 (36)	
Unknown	281 (82)	60 (18)		108 (77)	32 (23)		80 (58)	57 (42)	
Smoking status			<0.001			0.001			0.270
Never smoker	2446 (93)	189 (7)		1039 (86)	167 (14)		954 (69)	435 (31)	
Ex‐smoker	2350 (90)	255 (10)		1020 (80)	249 (20)		995 (66)	511 (34)	
Active smoker	933 (89)	114 (11)		436 (84)	86 (16)		517 (68)	238 (32)	
Unknown	545 (85)	98 (15)		189 (78)	53 (22)		168 (57)	126 (43)	
CCI score			<0.001			<0.001			<0.001
0	4185 (93)	307 (7)		1844 (86)	312 (14)		1957 (72)	771 (28)	
1	604 (85)	105 (15)		262 (82)	59 (18)		235 (61)	153 (39)	
2	834 (87)	121 (13)		358 (80)	92 (20)		273 (54)	234 (46)	
3+	651 (84)	123 (16)		220 (71)	92 (29)		169 (53)	152 (47)	
Highest educational level^b^			<0.001			<0.001			<0.001
Short	1957 (88)	271 (12)		786 (77)	240 (23)		807 (60)	534 (40)	
Medium	2863 (92)	262 (8)		1289 (85)	225 (15)		1264 (70)	551 (30)	
Long	1280 (93)	101 (7)		549 (87)	81 (13)		502 (74)	174 (26)	
Unknown	174 (89)	22 (11)		60 (87)	9 (13)		61 (54)	51 (46)	
Annual household income			<0.001			<0.001			<0.001
1st quartile	1342 (87)	203 (13)		557 (74)	197 (26)		502 (55)	416 (45)	
2nd quartile	1496 (88)	200 (12)		547 (78)	158 (22)		565 (61)	356 (39)	
3rd quartile	1643 (92)	137 (8)		698 (86)	112 (14)		733 (71)	303 (29)	
4th quartile	1775 (94)	110 (6)		872 (91)	<90 (9)		819 (78)	226 (22)	
Unknown	18 (75)	6 (25)		10 (67)	<5 (33)		15 (62)	9 (38)	
Cohabitation status			<0.001			<0.001			<0.001
Cohabiting	4168 (92)	350 (8)		1868 (86)	315 (14)		1834 (71)	742 (29)	
Living alone	<2105 (87)	<310 (13)		<815 (77)	<240 (23)		793 (58)	563 (42)	
Unknown	<5 (50)	<5 (50)		<5 (50)	<5 (50)		7 (50)	5 (50)	
pT4 tumour			<0.001			<0.001			<0.001
No	5553 (92)	469 (8)		2455 (84)	452 (16)		2543 (68)	1185 (32)	
Yes	721 (79)	187 (21)		229 (69)	103 (31)		91 (42)	125 (58)	
cM category			<0.001			<0.001			<0.001
cM0	5686 (91)	540 (9)		2464 (84)	458 (16)		2421 (68)	1138 (32)	
cM1	541 (83)	111 (17)		208 (70)	90 (30)		189 (54)	161 (46)	
Unknown	47 (90)	5 (10)		12 (63)	7 (37)		24 (69)	11 (31)	
MDT conference			0.031			0.253			0.061
Yes	3274 (90)	374 (10)		2256 (83)	475 (17)		2309 (67)	1149 (33)	
No	2463 (91)	233 (9)		205 (80)	52 (20)		134 (61)	87 (39)	
Unknown	537 (92)	49 (8)		223 (89)	28 (11)		191 (72)	74 (28)	
Neoadjuvant radio‐chemotherapy						<0.001			<0.001
No	Not relevant	Not relevant		2519 (84)	483 (16)		2052 (70)	889 (30)	
Yes	Not relevant	Not relevant		165 (70)	72 (30)		582 (58)	421 (42)	
Creation of temporary stoma									
No	6202			1731			327		
Yes	71			953			2307		
Peroperative perforation			<0.001			<0.001			<0.001
No	6187 (91)	593 (9)		2639 ()	526 (83)		2596 (68)	1202 (32)	
Yes	<85 (57)	<65 (43)		45 (61)	29 (39)		<40 (27)	<110 (73)	
Unknown	<5 (50)	<5 (50)					<5 (50)	<5 (50)	
Peroperative carcinosis found			<0.001			0.211			0.004
No	3597 (91)	360 (9)		1463 (83)	304 (17)		1386 (66)	719 (34)	
Yes	13 (52)	12 (48)		<5 (50)	<5 (50)		<5 (33)	<10 (67)	
Missing	2664 (90)	284 (10)		<1220 (83)	<250 (17)		<1250 (68)	<590 (32)	
Peroperative blood loss			<0.001			<0.001			<0.001
<500 mL	5958 (91)	563 (9)		2511 (84)	488 (16)		2423 (68)	1139 (32)	
≥ 500 mL	184 (73)	68 (27)		140 (72)	54 (28)		180 (57)	135 (43)	
Unknown	132 (84)	25 (16)		33 (72)	13 (28)		31 (46)	36 (54)	

*Note*: Values are *n* (%). To avoid showing identifiable data, some numbers have been changed to <N. *p*‐value was calculated using Pearson's chi‐square test of association or Fisher's exact test when appropriate only on non‐missing categories.

Abbreviations: ASA, American Society of Anesthesiologists; CCI, Charlson Comorbidity Index; MDT, multidisciplinary team; WHO, World Health Organization.

^a^ One unit = 12 g of pure ethanol.

^b^ International Standard Classification of Education (ISCED) 2011.

**FIGURE 2:**
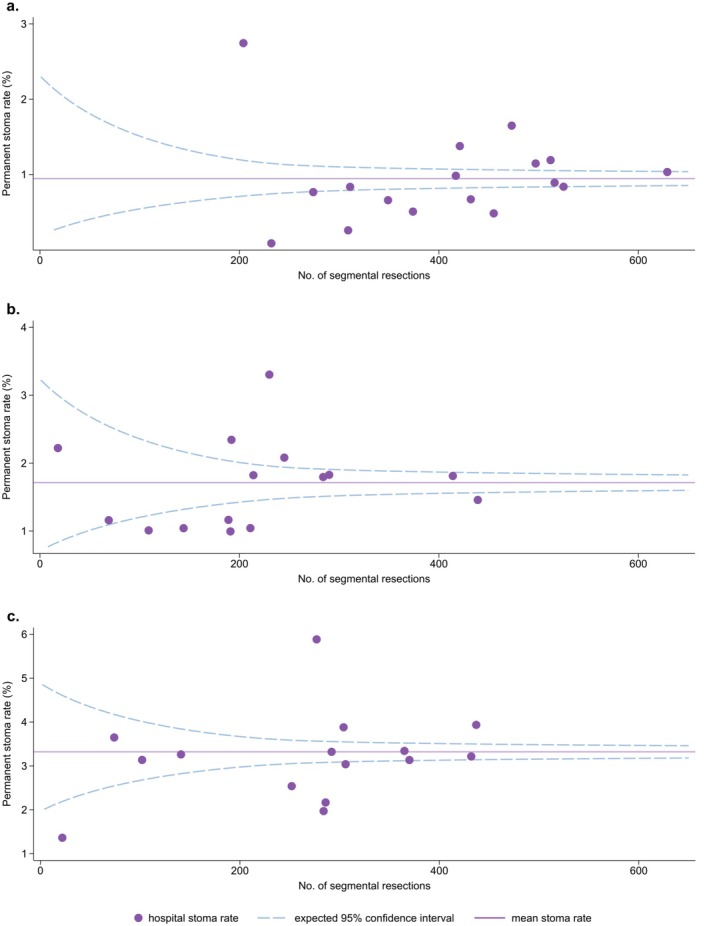
Rate of permanent stoma, stratified according to hospital and according to the number of segmental resections of cancer in the sigmoid colon (A), upper rectum (B) and mid‐rectum (C). Each dot represent one hospital, with only 15 hospitals treating rectal cancer.

The mean rate of placement of a permanent stoma in the sigmoid colon was 9%, with a variation between hospitals of 1% (95% CI 0.2‐3%) and 27% (95% CI 22‐34%) (Figure 2A). For the upper rectum, the mean rate of placement of a permanent stoma was 17%, with variation between hospitals of 10–33% (95% CI: 6–15% and 27–39%) (Figure 2B). For the mid‐rectum, the mean rate of placement of a permanent stoma was 33%, with variation between hospitals of 14–59% (95% CI: 4–36% and 53–65%) (Figure 2C).

**FIGURE 3:**
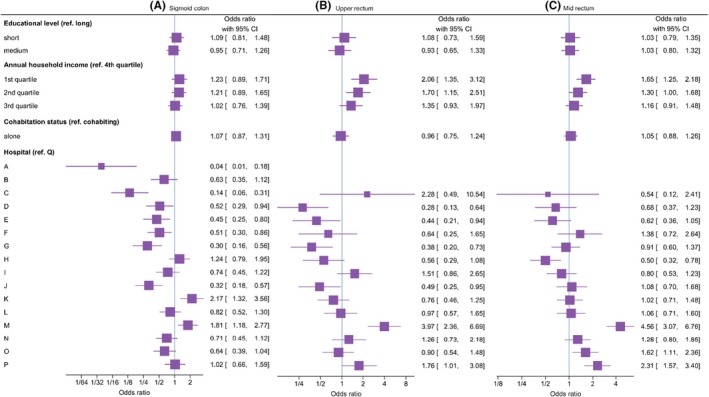
Forest plots of the association of permanent stoma with socio‐economic factors and between‐hospital variations in elective segmental resection of the sigmoid colon (A), upper rectum (B) and mid‐rectum (C). Hospitals A and B only treated colon cancer. Adjusted for demographics, lifestyle, comorbidity and perioperative risk factors. ref., reference.

The investigated predictors are shown, after adjustment, in **Figure** **3**. For the sigmoid colon, socio‐economic factors were not associated with the creation of a permanent stoma. Even after adjustment, a significant between‐hospital variation was found (*p* < 0.01), with Hospitals K and M having significantly higher odds (ORs of 2.17 and 1.81, respectively) of creating permanent stomas than the referent (Hospital Q), while Hospitals A and C had lower odds (ORs of 0.04 and 0.14, respectively) than the referent (**Figure** **3** **A**). For the upper rectum, the bottom two quartiles of household income were predictors of a permanent stoma after adjustment, and significant variations between hospitals were also present (*p* < 0.01), with an OR of up to 4.00 (Hospital M) and as low as 0.30 (Hospital D) compared with the referent (**Figure** **3** **B**). For mid‐rectal cancer, we found the lower quartile of household income a predictor of permanent stoma after adjustment, and marked variation was found between hospitals (*p* < 0.01), from an OR of 0.50 (Hospital H) to an OR of 4.60 (Hospital M) compared with the referent (**Figure** **3** **C**). Full details of the multivariable logistic regressions are provided in Table 2.

**TABLE 2:** Multivariable logistic regression of permanent stoma in resections for cancer in sigmoid colon, upper rectum and mid‐rectum, using resection with anastomosis as the reference level for comparison.

Variable	Sigmoid colon		Upper rectum		Mid‐rectum	
	OR (95% CI)	*p*	OR (95% CI)	*p*	OR (95% CI)	*p*
Gender (ref. female)						
Male	1.3 (1.02–1.55)	0.031	1.3 (0.98–1.64)	0.073	1.1 (0.96–1.38)	0.114
Age group (years) (ref. 50–64)						
<50	1.4 (0.74–2.54)	0.309	1.4 (0.70–2.90)	0.329	0.8 (0.52–1.29)	0.384
65–74	1.1 (0.81–1.47)	0.557	1.6 (1.11–2.29)	0.011	1.7 (1.35–2.08)	<0.001
75–84	2.2 (1.59–2.93)	<0.001	5.4 (3.71–7.86)	<0.001	5.5 (4.31–7.13)	<0.001
85+	6.6 (4.50–9.70)	<0.001	35.9 (20.39–63.31)	<0.001	60.3 (32.45–111.95)	<0.001
ASA score (ref. I)						
II	1.3 (0.93–1.78)	0.124	2.0 (1.38–2.98)	<0.001	1.2 (0.95–1.47)	0.143
III	3.5 (2.44–5.07)	<0.001	5.2 (3.30–8.24)	<0.001	2.4 (1.78–3.29)	<0.001
IV + V	10.3 (5.09–20.76)	<0.001	5.5 (1.21–24.98)	0.027	19.1 (2.40–152.41)	0.005
Unknown	1.4 (0.57–3.26)	0.495	2.6 (0.65–10.34)	0.176	0.9 (0.34–2.36)	0.824
WHO performance score (ref. 0)						
1	2.1 (1.58–2.89)	<0.001	1.6 (1.08–2.23)	0.018	1.6 (1.22–2.15)	0.001
2	3.9 (2.63–5.86)	<0.001	3.5 (1.86–6.63)	<0.001	2.4 (1.32–4.51)	0.004
3 + 4	5.1 (2.64–9.98)	<0.001	2.7 (0.71–10.05)	0.144	7.7 (1.94–30.80)	0.004
Unknown	1.0 (0.49–2.16)	0.935	1.2 (0.77–1.87)	0.412	1.1 (0.78–1.49)	0.634
Alcohol consumed per week (units^a^) (ref. 0–14)						
>14	0.8 (0.61–1.14)	0.259	1.3 (0.91–1.78)	0.165	0.8 (0.59–0.97)	0.027
Unknown	0.9 (0.59–1.47)	0.757	1.6 (0.89–2.85)	0.114	1.1 (0.69–1.77)	0.682
WHO body mass index (ref. normal weight)						
Underweight	1.3 (0.71–2.39)	0.386	2.1 (1.02–4.50)	0.043	1.4 (0.78–2.39)	0.273
Overweight	0.9 (0.72–1.13)	0.362	1.1 (0.81–1.37)	0.703	1.1 (0.91–1.33)	0.329
Obese	0.8 (0.61–1.07)	0.134	1.0 (0.68–1.35)	0.795	1.6 (1.26–2.05)	0.000
Unknown	1.4 (0.90–2.27)	0.125	1.2 (0.63–2.14)	0.635	1.9 (1.16–3.13)	0.011
Smoking status (ref. non‐smoker)						
Ex‐smoker	1.3 (1.01–1.61)	0.042	1.2 (0.93–1.59)	0.161	0.9 (0.76–1.12)	0.408
Active smoker	1.6 (1.20–2.17)	0.001	1.1 (0.80–1.64)	0.451	1.1 (0.83–1.35)	0.664
Unknown	1.9 (1.23–2.98)	0.004	1.3 (0.73–2.39)	0.350	1.3 (0.80–1.99)	0.316
Comorbidity (ref. not present)						
Cardiovascular disease	0.9 (0.72–1.21)	0.606	1.0 (0.73–1.31)	0.873	1.1 (0.91–1.35)	0.306
Chronic pulmonary disease	1.1 (0.90–1.36)	0.352	0.9 (0.73–1.23)	0.683	1.0 (0.80–1.19)	0.798
Diabetes	1.1 (0.91–1.40)	0.284	1.3 (0.98–1.72)	0.071	1.1 (0.90–1.38)	0.328
Dementia	1.6 (0.93–2.90)	0.086	1.0 (0.43–2.31)	0.991	1.0 (0.51–1.80)	0.886
Liver disease	1.7 (0.90–3.11)	0.104	1.1 (0.42–3.06)	0.797	1.3 (0.63–2.74)	0.459
Kidney disease	1.8 (1.24–2.74)	0.003	1.4 (0.78–2.36)	0.286	1.6 (0.91–2.90)	0.104
Nerve disease	1.0 (0.58–1.71)	0.990	1.3 (0.61–2.76)	0.505	1.8 (1.01–3.28)	0.045
Other cancer	1.2 (0.93–1.48)	0.169	1.0 (0.79–1.40)	0.748	1.7 (1.34–2.09)	<0.001
Connective tissue disease	1.2 (0.91–1.56)	0.213	1.2 (0.87–1.78)	0.225	1.0 (0.78–1.39)	0.767
**Affective disorder	0.9 (0.71–1.15)	0.415	1.2 (0.93–1.66)	0.145	1.2 (0.96–1.50)	0.118
Schizophrenia spectrum disorder	1.7 (0.56–5.13)	0.346	0.7 (0.15–2.85)	0.579	1.9 (0.57–6.15)	0.297
Disorder of adult personality and behaviour	1.5 (0.38–6.14)	0.556	0.6 (0.06–5.01)	0.609	1.1 (0.25–4.47)	0.946
Psychoactive drug abuse disorder	1.1 (0.80–1.64)	0.448	1.3 (0.79–1.99)	0.337	1.5 (1.05–2.12)	0.024
Highest educational level (ref. long)						
Short	1.1 (0.81–1.48)	0.568	1.1 (0.73–1.59)	0.695	1.0 (0.79–1.36)	0.810
Medium	0.9 (0.71–1.26)	0.713	0.9 (0.65–1.33)	0.678	1.0 (0.80–1.32)	0.812
Unknown or unclassified	0.8 (0.45–1.45)	0.477	0.3 (0.12–0.85)	0.023	1.2 (0.72–2.10)	0.457
Annual household income (ref. 4th quartile)						
1st quartile	1.2 (0.89–1.71)	0.205	2.1 (1.36–3.13)	0.001	1.7 (1.25–2.18)	<0.001
2nd quartile	1.2 (0.89–1.65)	0.220	1.7 (1.16–2.51)	0.007	1.3 (1.00–1.69)	0.050
3rd quartile	1.0 (0.76–1.39)	0.875	1.4 (0.94–1.98)	0.102	1.2 (0.91–1.48)	0.217
Unknown	3.1 (0.83–11.79)	0.093	4.0 (0.39–40.69)	0.247	2.6 (0.68–9.86)	0.166
Cohabitation status (ref. cohabiting)						
Living alone	1.1 (0.87–1.31)	0.539	1.0 (0.75–1.24)	0.789	1.1 (0.88–1.26)	0.570
Unknown	2.4 (0.17–32.97)	0.515	4.5 (0.14–146.68)	0.394	0.9 (0.14–6.36)	0.938
pT4 tumour (ref. no)						
Yes	2.1 (1.64–2.61)	<0.001	2.0 (1.41–2.78)	<0.001	2.5 (1.74–3.50)	<0.001
cM category (ref. cM0)						
cM1	1.8 (1.38–2.41)	<0.001	2.3 (1.61–3.18)	<0.001	1.7 (1.30–2.24)	<0.001
Unknown	1.2 (0.44–3.42)	0.701	1.9 (0.53–6.64)	0.325	0.9 (0.38–2.14)	0.805
MDT conference (ref. yes)						
No	1.0 (0.76–1.26)	0.884	1.4 (0.94–2.21)	0.094	1.1 (0.74–1.57)	0.680
Unknown	0.6 (0.37–1.00)	0.048	0.4 (0.20–0.65)	0.001	0.7 (0.44–1.01)	0.055
Neoadjuvant radio‐chemotherapy (ref. no)						
Yes			3.1 (2.13–4.59)	<0.001	2.3 (1.90–2.80)	<0.001
Peroperative perforation (ref. no)						
Yes	8.1 (5.26–12.49)	<0.001	3.0 (1.61–5.59)	0.001	5.4 (3.48–8.49)	<0.001
Unknown	4.6 (0.38–55.49)	0.227			10.3 (0.27–384.73)	0.208
Peroperative carcinosis found (ref. no)						
Yes	5.0 (1.71–14.37)	0.003				
Missing	1.4 (0.63–2.92)	0.443				
Peroperative blood loss (ref. no)						
≥ 500 mL	2.6 (1.77–3.70)	<0.001	1.9 (1.26–2.93)	0.002	1.6 (1.22–2.17)	0.001
Unknown	1.5 (0.86–2.46)	0.161	1.4 (0.62–3.34)	0.401	2.1 (1.16–3.76)	0.015
Year of diagnosis	1.0 (0.96–1.11)	0.419	1.0 (0.96–1.13)	0.316	1.1 (1.01–1.13)	0.026
Hospital (ref. Q)						
A	0.0 (0.01–0.18)	<0.001				
B	0.6 (0.35–1.12)	0.118				
C	0.1 (0.06–0.31)	<0.001	2.3 (0.49–10.37)	0.297	0.5 (0.12–2.43)	0.426
D	0.5 (0.29–0.94)	0.030	0.3 (0.12–0.63)	0.002	0.7 (0.37–1.23)	0.199
E	0.5 (0.25–0.80)	0.007	0.4 (0.20–0.90)	0.025	0.6 (0.38–1.07)	0.086
F	0.5 (0.30–0.86)	0.011	0.6 (0.25–1.64)	0.353	1.4 (0.72–2.64)	0.331
G	0.3 (0.16–0.56)	<0.001	0.4 (0.19–0.73)	0.004	0.9 (0.60–1.36)	0.640
H	1.2 (0.79–1.95)	0.343	0.6 (0.28–1.07)	0.078	0.5 (0.32–0.78)	0.002
I	0.7 (0.45–1.22)	0.233	1.5 (0.86–2.63)	0.153	0.8 (0.52–1.23)	0.315
J	0.3 (0.18–0.57)	<0.001	0.5 (0.25–0.95)	0.036	1.1 (0.70–1.67)	0.732
K	2.2 (1.32–3.56)	0.002	0.8 (0.46–1.24)	0.271	1.0 (0.71–1.49)	0.867
L	0.8 (0.52–1.30)	0.401	1.0 (0.56–1.62)	0.855	1.1 (0.71–1.61)	0.747
M	1.8 (1.18–2.77)	0.006	3.9 (2.35–6.65)	<0.001	4.6 (3.08–6.76)	<0.001
N	0.7 (0.45–1.12)	0.139	1.3 (0.73–2.17)	0.405	1.3 (0.89–1.85)	0.187
O	0.6 (0.39–1.04)	0.073	0.9 (0.54–1.47)	0.652	1.6 (1.12–2.37)	0.012
P	1.0 (0.66–1.59)	0.923	1.8 (1.02–3.10)	0.043	2.3 (1.57–3.40)	<0.001
Intercept	0.0 (0.01–0.03)	< 0.001	0.0 (0.00–0.02)	<0.001	0.0 (0.01–0.05)	<0.001

*Note*: Goodness‐of‐fit test: (i) 0.587 for sigmoid colon; (ii) 0.081 for upper rectum; (iii) 0.075 for mid rectum.

Abbreviations: ASA, American Society of Anesthesiologists; MDT, multidisciplinary team; ref., reference; WHO, World Health Organization.

^a^ One unit = 12 g of pure ethanol.

^b^ International Standard Classification of Education (ISCED) 2011 [13].

Post‐hoc analyses with reduction of hospitals to tertiles (low, medium or high volume) of total patient volume showed clinically significant odds ratios for low (OR = 0.38, 95% CI: 0.29–0.50) and medium (OR = 0.81, 95% CI: 0.66–0.99) volume hospitals compared with high‐volume hospitals for sigmoid colon. For the upper third of the rectum, a significant odds ratio (OR = 0.46, 95% CI: 0.27–0.78) was found only for hospitals with a low patient volume compared with those with a high patient volume, while for the mid third of rectum the patient volume was not significant (not shown in the table).

We also checked in the NPR if patients had their stoma reversed within the first year after surgery; during this time period, only 3% of the patients registered with a permanent stoma had it reversed (data not shown in tables).

## DISCUSSION

The aim of this study was to explore whether socio‐economic factors and the treating hospital were predictors of creation of a permanent stoma in elective segmental resections for CRC with curative intent. We sought to investigate whether variations reported in recent annual reports persisted when accounting for patient case mix, extent of disease and various intra‐operative factors. Our focus was on common tumour locations where the surgical approach is considered relatively simple and standardized: the right colon, the sigmoid colon, the upper third of the rectum and the mid third of the rectum. Our objective was not to set a target or indicator standard for the proportion of permanent stomas in these resections but to compare the proportion of stomas after adjustment for the forementioned factors.

A limited number of permanent stomas was observed in right‐sided resections, precluding detailed analyses. For resections of the rectum, however, low household income was associated with an increased risk of approximately 1.7‐fold and 2.1‐fold of a permanent stoma in the upper rectum and the mid third of the rectum, respectively. Education level and living alone showed no correlation in any of the groups. Clear variations between hospitals persisted, which were unexplained by the included covariates.

The odds ratios for socio‐economic factors should be contextualized appropriately. Adjusted for various factors associated with permanent stoma, the odds ratios for many covariates far exceeded those for socio‐economics factors (Table 2). Therefore, the odds ratios of socio‐economic factors represent only the direct effect and are not the strongest predictors of outcome. While these variations align with expectations based on other studies linking socio‐economic factors to unfavourable outcomes in CRC [23, 24, 25], they are not the desired outcome. Clearly, some hospitals exhibited higher and lower odds ratios for permanent stoma within the same segment of total patient volume (**Figure** **3**), with Hospital M consistently having higher odds ratios across all three groups. Together with Hospital K, Hospital M is the only hospital in which locally advanced rectal cancer is treated. Variations between hospitals are not surprising and have been highlighted by the DCCG in several annual reports over the years. A number of reasons for these variations have been suggested, such as different case mix, proportions of neoadjuvant radiotherapy and locally advanced rectal cancer. However, despite adjustment for these variations, including socio‐economic factors, these differences persisted in the present study.

One might speculate that hospitals with a low proportion of patients treated with a permanent stoma could have a higher proportion of construction of temporary protective stomas, but such an association was not found (data not shown).

Comparable studies in the existing literature are mainly from countries with different healthcare systems (e.g. the United States) or are older studies. Our findings, particularly that certain high‐volume hospitals have elevated stoma rates, deviated from our expectations. A Dutch study from 2021 found no association between hospital volume and rates of sphincter‐preserving surgery, with variations between hospitals for such surgery of 38–98% for the upper rectum and 65–100% for the mid‐rectum [26], while a Swedish study reported non‐reversed stoma rates varying from 18% to 29% between regions [27]. Some systematic reviews concluded that hospital volume was inversely related to the rate of permanent stomas, but these included only a few older European studies [28, 29].

One limitation of the present study is an inability to adjust for expected poor functional outcomes. Ng et al. reported that the prevalence of faecal incontinence in elderly patients may be as high as 15% [30]. In these cases, the choice of a permanent stoma (either iAPE or Hartmann's procedure) may be appropriate [2, 31]. We had no information about the patients' pre‐existing incontinence problems, which are often only disclosed during the preoperative consultation. While we do not suspect that faecal incontinence varies significantly between hospitals, it may impact the association of socio‐economic factors with a permanent stoma.

A second limitation is the potential effect of patient preferences, which are likely to influence the rate of construction of permanent stomas in borderline cases [32]. As stated earlier, the conflicting evidence on quality of life with a permanent stoma compared with an anastomosis complicates matters, and we lack data on patient preferences. Third, the cohort's age extends up to 2018, raising the possibility of changes in treatment practices since then. However, as indicated earlier, recent reports continue to show between‐hospital differences, suggesting a persistent pattern. Fourth, we had no data on ethnicity or religion, which could potentially influence patient preferences. Fifth, the register setup only allows examination of possible association patterns, not necessarily causal relations. Finally, despite adjusting for a wide range of comorbidities and other known risk factors, we cannot rule out that registration errors, missing data, possible overadjustment and residual confounding may have influenced the results. Most of these potential limitations are inherent to the use of register data and beyond our control.

The DCCG database contains a single variable with information on whether an anastomosis was, or was not, created during surgery. However, we did not use this variable in the present study because >99% of information in the variable were unknown or undefined in the years 2009–2013. Therefore, we mainly relied on the surgical procedure codes which imply whether a patient had a stoma or an anastomosis, as described in the Method section. A validation study by Klein et al. [12] found high accuracy in permanent stoma rates from a random sample of patients from 2014 to 2017, and we are thus confident that our data on whether the patient had a stoma or an anastomosis are reliable.

Possible collinearity could be present between some of the adjustment covariates, but this is of minor importance because the population is very large, the methods are robust with respect to collinearity and the effect of the covariates are not of importance in this study. Also, collinearity could be present, to some degree, between the main predictors. However, interpreting these factors one by one, without accounting for the others, would make interpretation of the results difficult.

Despite these limitations, the strengths of this study lie in the prospectively collected register data from multiple sources, facilitating the comparison of a large number of cases and highlighting systematic differences, which are often challenging to detect in annual reports. This approach reduces the risk of selection bias. The various data sources also enable adjustment for a wide range of known confounders.

### Perspectives

The findings of this study underline the need for further exploration of inequalities and disparities in clinical practice across hospitals. We recommend directing attention towards the consultation process, a complex interaction between the healthcare professional and the patient newly diagnosed with a serious disease. The evidence base for different treatment options, the manner of presenting these choices to the patient, the patient's health literacy and personal resources, and the surgeon's attitude all contribute to the final decision. We advocate for the implementation of a formalized context of shared decision‐making as the optimal approach to ensure personalized decisions for the individual patient. We propose that this is explored in greater detail. It should be kept in mind that the odds ratios of other known risk factors far exceeded those of the socio‐economic factors.

## CONCLUSION

Our study revealed an association between low household income and the likelihood of having a permanent stoma during rectal cancer surgery in the upper two‐thirds of the rectum. This association was not observed with educational level or cohabitation status. Furthermore, even after adjusting for multiple variables, we identified significant between‐hospital variations in the risk of having a permanent stoma following a sigmoid or a rectal resection for CRC.

## AUTHOR CONTRIBUTIONS

**Erik Frostberg:** Conceptualization; data curation; funding acquisition; writing – review and editing. **Hans Bjarke Rahr:** Conceptualization; funding acquisition; writing – review and editing. **Sören Möller:** Conceptualization; formal analysis; funding acquisition; methodology; writing – review and editing. **Søren Rattenborg:** Conceptualization; data curation; formal analysis; funding acquisition; methodology; project administration; visualization; writing – original draft; writing – review and editing.

## FUNDING INFORMATION

This study was funded by the Region of Southern Denmark, Lillebaelt Hospital, Denmark and Dagmar Marshalls Fund, Denmark. The funders were not involved in the research.

## CONFLICT OF INTEREST STATEMENT

The authors report no conflicts of interest.

## ETHICS STATEMENT

This study received approval from the DCCG and Danish Clinical Quality Program (DCCG‐2018‐03‐08a) and from the Danish Data Protection Agency (jr. no. 18/15252).

## PATIENT CONSENT STATEMENT

In accordance with Danish law, patient consent was not necessary for this register‐based study.

## Data Availability

The authors have full access to the study data used in this publication, but data cannot be made available according to Danish legislation.
